# Removal of Parabens from Aqueous Solution Using β-Cyclodextrin Cross-Linked Polymer

**DOI:** 10.3390/ijms11092459

**Published:** 2010-09-20

**Authors:** Yuk Ping Chin, Sharifah Mohamad, Mhd Radzi Bin Abas

**Affiliations:** Environmental Research Group, Department of Chemistry, Faculty of Science, University of Malaya, 50603 Kuala Lumpur, Malaysia; E-Mails: ping1908@yahoo.com (Y.P.C.); radzi@um.edu.my (M.R.B.A.)

**Keywords:** β-cyclodextrin polymer, parabens, adsorption

## Abstract

The removal of four parabens, methyl-, ethyl-, propyl-, and benzyl-paraben, by β-cyclodextrin (β-CD) polymer from aqueous solution was studied. Different β-CD polymers were prepared by using two cross-linkers, *i.e.*, hexamethylene diisocyanate (HMDI) and toluene-2,6-diisocyanate (TDI), with various molar ratios of cross-linker. β-CD-HMDI polymer with molar ratio of 1:7 and β-CD-TDI polymer with ratio 1:4 gave the highest adsorption of parabens among the β-CD-HMDI and β-CD-TDI series, and were subsequently used for further studies. The adsorption capacity of β-CD-HMDI is 0.0305, 0.0376, 0.1854 and 0.3026 mmol/g for methyl-, ethyl-, propyl-, and benzyl-paraben, respectively. β-CD-TDI have higher adsorption capacities compared with β-CD-HMDI, the adsorption capacity are 0.1019, 0.1286, 0.2551, and 0.3699 mmol/g methyl-, ethyl-, propyl-, and benzyl-paraben respectively. The parameters studied were adsorption capacity, water retention, and reusability. Role of both cross-linker in adsorption, hydrophobicity of polymers, and adsorption capacity of different parabens were compared and discussed. All experiments were conducted in batch adsorption technique. These polymers were applied to real samples and showed positive results.

## 1. Introduction

Since their development for use in personal care products in the 1920’s, parabens are the most popular and widely used preservative in food, pharmaceutical, and cosmetic products [1] due to their low toxicity, low cost, and broad spectrum of antimicrobial activity [2]. However, a small percentage of the general population suffers from paraben allergies [3]. Recent studies have also shown that parabens possess estrogen agonist activity [4] and have been detected in human breast tumor tissue with an average concentration of 20 ng/g of tissue [5]. Parabens may also cause male infertility as they may cause testis mitochondrial dysfunction [6]. Due to their potential impacts on human health, many countries have introduced legislations that govern the use of parabens. For example, the maximum authorized concentration (MAC) of individual parabens in cosmetics is set at 0.4% in China and 1.0% in Japan. The Council Directive 76/768/EC of the European Community meanwhile restricts the preservation of cosmetic products with methyl, ethyl, propyl, butyl, and benzyl paraben (MP, EP, PP, BuP, and BP) to a MAC of 0.4% (w/w) for single parabens and 0.8% (w/w) for paraben mixtures [7].

Although removal of parabens in the treatment plant is efficient [8], they can still be found in various types of water [9–13]. Methods such as photosensitized degradation [14], ozonation [15], and chlorine dioxide treatment [8] give a high percentage of removal but they produce disinfection by-products (DBPs)[16]. For example, chlorine dioxide treatment can produce chlorite (ClO_2_^−^) and chlorate (Cl_2_O_2_), which are potentially toxic [16]. Hence, the adsorption method may offer a much safer way for removing parabens from water and wastewaters since it is a method free of harmful substances, and is environmentally friendlier, has a low initial cost, flexible and simple in design and operation [17,18].

Much attention has been focused recently on the use of biopolymers and natural molecules as adsorbents [17]. Among these are natural polymers such as chitin [19,20], chitosan [21,22], starch [23–25], and cyclodextrin [26–28]. Cylodextrins have gained prominence in recent years because their cavity, which is hydrophobic in nature, can entrap different kinds of compounds including organic, inorganic, organometallic and metaloorganic, which may be neutral, cationic, anionic, or even radical [29]. Moreover, cyclodextrin has low toxicity, excellent biocompatibility [30] and biodegradable [31], and is harmless to humans and environment [32]. Another advantage is that the production of cyclodextrins is environmentally friendly since they can be obtained by enzymatic degradation of starch [33]. There are three main types of cyclodextrins: α-, β-, and γ-cyclodextrin, which contain six, seven, and eight glucopyranose units that are linked together by α-1,4-glycosidic bonds [34]. Among these three types, β-CD is most accessible and low-priced [35]. However, its solubility in water makes it inconvenient to use as adsorbent [36], therefore, β-CD polymer may offer better alternatives.

Various applications of β-CD polymer have been reported in the literature, for example, the removal of phenol from wastewater [37], extraction of steroidal compounds [38], solid-phase extraction material for the analysis of carcinogenic aromatic amines [39], fluorescence sensor for direct determination of bisphenol A [40], extraction of Co(II)[41], *etc*. In view of the fact that parabens are able to form inclusion complex with β-CD [42–44], we are investigating the efficiency of β-CD polymer as an adsorbent for the removal of parabens from various matrices. To the best of our knowledge, studies on the removal of parabens from aqueous solution using β-CD polymer as an adsorbent have yet to be reported. The effects of various experimental parameters such as types and ratio of cross-linkers, water retention, and reusability of polymer are presented and discussed.

## 2. Results and Discussion

### 2.1. Characterization of β-CD-HMDI and β-CD-TDI Polymer

**Figure 1:**
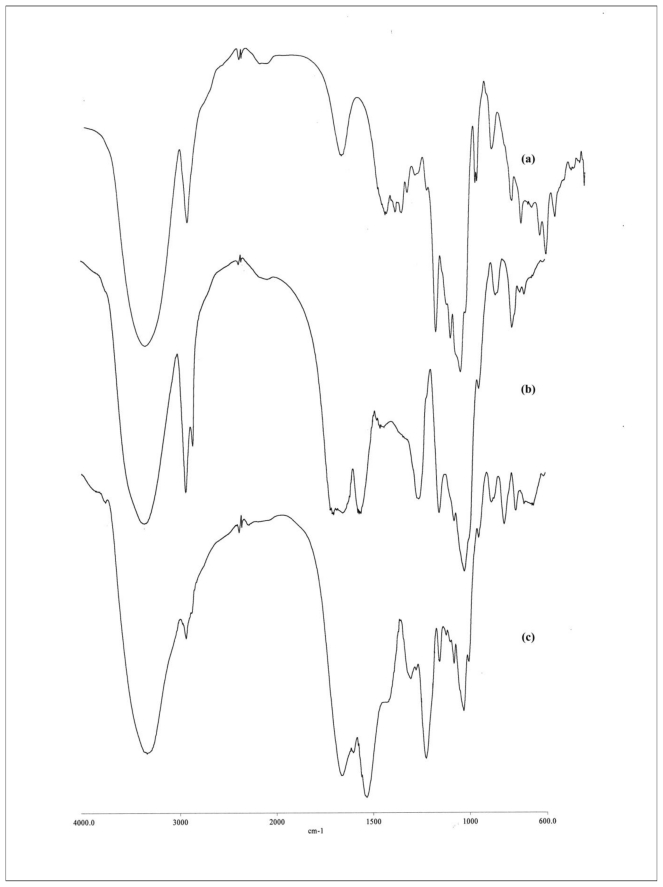
FT-IR spectra for (**a**) β-CD, (**b**) β-CD-HMDI, and (**c**) β-CD-TDI.

Figure 1a,b,c shows the FT-IR spectra of β-CD, β-CD-HMDI, and β-CD-TDI, respectively. The peak at 2270 cm^−1^ (corresponding to isocyanate group) is absent in both Figures 1b,c, indicating that the polymerization reaction is complete [39]. The strong bands at 2860 and 2934 cm^−1^ in Figure 1b correspond to the methylene group in HMDI, while the bands at 3364 and 1719 cm^−1^ represent the NH and C=O groups. The stretching of NHCO, which indicates the formation of carbamate group, *i.e.*, characteristic group of polymerization, is observed at 1570 cm^−1^. The bands at 1535 and 1603 cm^−1^ in Figure 1c represent the aromatic group in TDI. The FT-IR spectra obtain are similar to obtained by Bhaskar *et al.* [39]. Therefore, we conclude that polymerization between β-CD and cross-linker are completed and β-CD polymers are formed.

### 2.2. Effect of the Types and Amount of Cross-Linker

**Figure 2:**
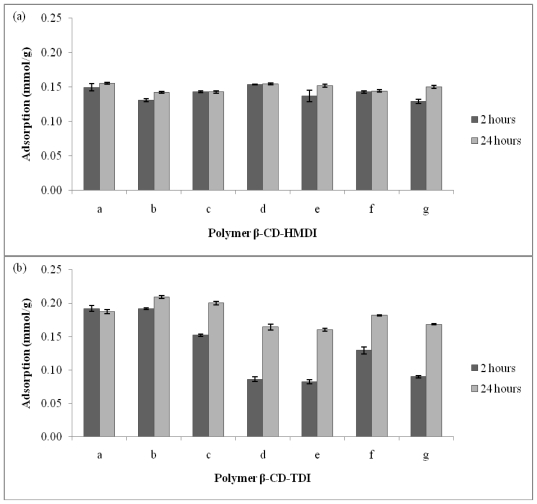
Comparison of adsorption (mmol/g) towards parabens by varying (**a**) β-CD-HMDI and (**b**) β-CD-TDI polymer (time: 2 hours and 24 hours; concentration: 10 ppm; volume: 5 mL; molar ratio of β-CD:crosslinker: 1:4(a), 1:7(b), 1:10(c), 1:13(d), 1:16(e), 1:19(f), and 1:12(g)).

In order to evaluate the influence of cross-linker on the adsorption and the rate of adsorption for parabens, tests were carried out using polymers with different molar ratios of β-CD:cross-linker and types of cross-linker, and at contact times of two and 24 hours. All β-CD polymers prepared showed good adsorption capacities towards parabens (Figure 2a,b). Figure 2a shows that increasing the amount of HMDI and different contact times did not affect the adsorption of parabens onto β-CD-HMDI very much. However, there is a marked difference in the adsorption of parabens onto β-CD-TDI, especially with a higher amount of TDI, and there appears to be a bigger difference between the adsorptions at contact times two and 24 hours (Figure 2b). The results show that the adsorption of parabens on β-CD polymers is dependent on the amount of TDI used in the preparation of the polymers, but not on the amount of HMDI. However, β-CD-HMDI(a) and β-CD-TDI(b) were chosen for further studies since they provide relatively higher adsorption capacities amongst each of their series. The two selected polymers are also economically more viable since they contain lower amounts of cross-linkers.

**Figure 3:**
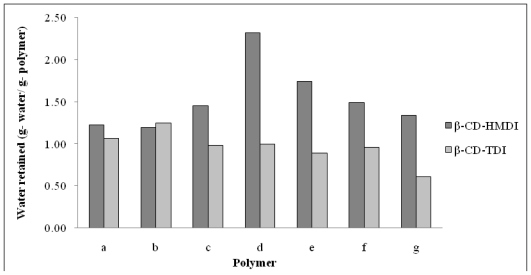
Water adsorption by β-CD-HMDI and β-CD-TDI polymer (molar ratio of β-CD:crosslinker: 1:4(a), 1:7(b), 1:10(c), 1:13(d), 1:16(e), 1:19(f), and 1:12(g)).

The water absorption property of all polymers were then examined (Figure 3). The results show that, in general, β-CD-HMDI polymers absorbed more water than the β-CD-TDI polymers. This is probably because TDI has poor contact with aqueous solution due the presence of an aromatic group in TDI, and that TDI, as a cross-linker, can form a more non-polar polymeric network compared to HMDI. As the polymer becomes more hydrophobic, the diffusion of water is less, and hence the contact between the polymer and solute is slower. If there is not enough swelling of the polymer network, large molecules cannot penetrate across the adsorbent-cross linker chain [45]. Therefore, the adsorption of parabens is expected to decrease when the amount of TDI is increased, except at the contact time of 24 hours (Figure 2b). However, results in Figures 2a,b and Table 1 show that the adsorption of parabens on β-CD-TDI polymer is higher than on β-CD-HMDI polymer. This anomaly will be discussed in Section 2.3.

### 2.3. Adsorption Behavior of Polymers toward Parabens

Table 1 shows the adsorption capacities of each paraben on β-CD-HMDI(a) and β-CD-TDI(b) polymers for single and mix solutions. In general, the adsorption capacities of each paraben in single solution are higher compared to the adsorption capacities of each paraben in mix solution. This is probably because in solution containing the mixture of parabens, there exists a competition among the four parabens to be adsorbed into a limited cavity. Adsorption capacity for β-CD-TDI(b) (0.555 mmol/g) was higher than β-CD-HMDI(a) (0.4080 mmol/g) as shown in Table 1. β-CD-TDI has higher adsorption capacity may due to their higher hydrophobicity compare to β-CD-HMDI.

When β-CD is immersed in an aqueous solution, the water molecules will fill in the apolar cyclodextrin’s cavity [34]. This polar-apolar interaction is energetically unfavorable [34]. Therefore, water molecules will be easily substituted by paraben molecules. Table 1 also shows that the adsorption capacity increases with decreasing of polarity of parabens in the order BP > PP > EP > MP (benzyl-, propyl-, ethyl-, and methyl-paraben, respectively). BP shows the highest removal either in single solution or a mixture of parabens (Table 1). This is because BP is the least polar and least soluble in water, therefore BP can easily adsorb into the cavity of β-CD due to the strongest solute-adsorbent interaction.

### 2.4. Reusability

**Figure 4:**
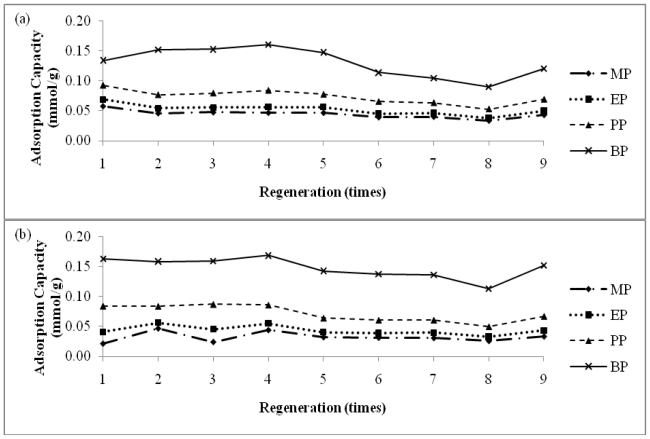
Adsorption capacity of regenerated (**a**) β-CD-HMDI(a) and (**b**) β-CD-TDI(b) towards parabens.

A good adsorbent is reusable and has high number of regeneration times. The reusability of an adsorbent can reduce the large amount of processing costs since adsorbent can be the most expensive consumable component in an experiment. Methods and materials used in the regeneration process are also important; they have to be simple and low cost. In this study, methanol was used since parabens have high solubility in methanol. Parabens can be desorbed from β-CD polymer by eluting 10 mL of methanol. The capacities of regenerated polymer are shown in Figure 4. The regenerated polymer could still achieve between 78% to 112% capacities after nine regenerations.

### 2.5. Application on Real Sample

Application of the prepared polymers on real samples shows very positive results, especially for PP and BP. The results are shown in Tables 2 and 3. In general, the results are consistent with the adsorption of parabens from mix solution.

## 3. Experimental Section

### 3.1. Reagents and Solutions

All reagents were used without further purification unless indicated otherwise. β-CD hydrate, 99% was purchased from Acros, Hungary. Methyl paraben (MP), ethyl paraben (EP), propyl paraben (PP), and benzyl paraben (BP) were supplied by Fluka, United Kingdom. Toluene-2,6-diisocyanate (TDI) (90%) was purchased from Fluka, Germany. 1,6-Hexamethylene diisocyanate (HMDI)(90%) was obtained from Aldrich, USA and pre-filtered methanol (HPLC grade) was supplied by Merck, Germany. *N*,*N*-Dimethylformamide (DMF) of analytical grade reagent from Fisher Scientific, UK. DMF was distilled before use. Single-solute stock solution of 100 mg/L was prepared in boiled ultrapure deionised water (Elga, USA). Mix-solute solution containing 100 mg/L each of MP, EP, PP, and BP was also prepared in the same manner. Working solutions were prepared daily by appropriate dilution of the stock solution.

### 3.2. Instrumentation

HPLC was used to determine the concentration of parabens before and after removal. High performance liquid chromatography (HPLC) system that used consist of a LC-20AT pump, a SPD-M20A diode array detector, a SIL-20AHT auto sampler, a CTO-20AC column oven and CBM-20A communication bus module (Shimadzu, Japan). The parabens were separated on a reversed-phase Chromolith RP-18 monolithic column (100 mm × 4.6 mm i.d., Merck, Germany). LC Solution Software (release 1.23 SPI) was used to monitor the LC system and data acquisition. The mobile phase consisted of methanol (eluent A) and water (eluent B), gradient elution: 0 (zero) minutes, A 50% and B 50%, 9 minutes, A 79.8% and B 20.2%. Separations were accomplished at a flow rate of 1.00 mL/min at 30 °C and injection volume of 20 μL, at detection wavelength of 254 nm.

### 3.3. Preparation of β-CD Polymer

β-CD polymers were prepared according to method of Bhaskar *et al.* [39]. Briefly, 10 g of β-CD was first dissolved 30 mL of dry DMF at room temperature. A calculated amount of cross-linker (HMDI or TDI) was then added dropwise and the mixture stirred for 4 hours at 70 °C. The polymer formed was then precipitated by the addition of excess methanol, filtered, washed with methanol and dried overnight in an oven at 70 °C. The dried polymer was first ground and sieved using a 53 μm sieve before used.

Different molar ratios of β-CD:cross-linker were studied and the polymer with the highest adsorption capacity towards parabens in each series was chosen for further investigation. The polymers were prepared in two batches; one using HMDI as the cross-linking agent and the other using TDI. Eight different molar ratios of β-CD:cross-linker were used for each batch, namely, 1:4(a), 1:7(b), 1:10(c), 1:13(d), 1:16(e), 1:19(f), and 1:12(g).

### 3.4. Characterization of Polymer

Fourier transform infrared spectroscopy (FT-IR) spectra of polymers were taken on a Perkin-Elmer RX1 FT-IR spectrometer from 400–4000 cm^−1^. The samples were prepared in pellet form using spectroscopic grade KBr powder.

Water retention property of the polymer was determined by packing it into an empty solid-phase extraction cartridge and dried in a desiccator for 24 hours. The cartridge was then attached to a vacuum manifold (Lichrolut, Merck, Germany) followed by the addition of ultrapure deionized water to soak the polymer. After soaking the polymer for 24 hours, the excess water was drained off at a flow rate of 2 mL/min. Weights of polymer before and after soaking was recorded. The average of triplicate was taken as show in Figure 3.

### 3.5. Removal of Parabens from Water

About 0.05 g of β-CD polymers was added into a flask containing solution of parabens mixture and the flask was shaken on an orbital shaker (YIHDER TS-560, Taiwan) at 150 rpm. The initial concentrations of parabens and shaking times were varied according to the parameters examined. The supernatant was withdrawn at pre-determined time intervals using a syringe fitted with a 0.2 μm pore size PTFE membrane filter. Three blank samples were treated in the same conditions for every bath of polymers. The adsorption capacity of parabens, *q*, is calculated as follows:

(1) $$q=\frac{(C_{0}-C)V}{m}$$

where *C*_0_ and *C* denote the initial concentration of solution before removal and concentration of solution at equilibrium, both solutions were determined by using HPLC as discussed in Section 3.2. V is the volume of solution and m is mass of adsorbent used.

### 3.6. Reusibility of β-CD Polymer as Adsorbent

To study how effective the recycled polymer was for paraben removal, 0.2 g of polymer was stirred in 100 mL of 100 ppm parabens mixture solution for 5 hours and filtered. The concentrations of parabens in the filtrate were determined by HPLC. The polymer was then dried in an oven at 70 °C overnight. After drying, 10 mL of methanol was used to elute the adsorbed parabens and the polymer again dried overnight at 70 °C. The experiment was repeated for 9 additional cycles using the same polymer.

### 3.7. Application of Polymer to Real Samples

The polymers were applied on 7 samples to determine their removal ability in different matrices. The samples can be divided into 2 categories, *i.e.*, water sample and commercial products. Water samples were tap water, bottled drinking water (purchased from hypermarket in Kuala Lumpur), swimming pool water (collected from the University of Malaya, Sports Centre’s swimming pool), river water (from a river flowing through the campus of University of Malaya), and treated wastewater effluent (from sewage treatment plant in Kuala Lumpur). 50 mL of each sample was used for the study. Soy sauce, and mouth rinse were used to represent commercial products. 1 mL/g of these samples was diluted by 50 mL of deionized water. Parabens found in the ingredient list of the commercialised samples except soy sauce.

All the samples were spiked with 100 μL of 100 mg/L parabens solution. Then, 0.05 g of polymer was added to the samples and was shaken overnight. Concentrations of parabens in the samples before and after removal were determined by HPLC.

## 4. Conclusions

This study indicates the suitability of β-CD cross-linked polymer to be used as adsorbent for the removal of parabens from aqueous solution. The adsorption capacity of β-CD-TDI polymer is higher than β-CD-HMDI polymer. In addition, the adsorption of parabens was dependent on the molar ratio of TDI but independent on the molar ratio of HMDI. Both polymers have high reusability and simple regeneration procedure. These polymers show a positive result when tested on real samples.

## Figures and Tables

**Table 1:** Adsorption capacities of β-CD-HMDI(a) and β-CD-TDI(b) in single solute solution and mix solution. (time: 24 hours; concentration: 100 ppm; volume: 50 mL). MP: methyl-paraben; EP: ethyl-paraben; PP: propyl-paraben; BP: benzyl-paraben.

Adsorbent	Paraben	Single solute solution		Mix solution	
		Adsorption capacity (mmol/g)	R.S.D.(%) n = 3	Adsorption capacity (mmol/g)	R.S.D.(%) n = 3
β-CD-HMDI(a)	MP	0.0305	9.08	0.0239	7.51
	EP	0.0376	9.36	0.0357	7.56
	PP	0.1854	5.67	0.0715	7.33
	BP	0.3026	8.10	0.2769	7.06
	Total			0.4080	
β-CD-TDI(b)	MP	0.1019	3.03	0.0324	5.42
	EP	0.1286	3.22	0.0508	6.31
	PP	0.2551	3.04	0.1150	2.73
	BP	0.3699	3.44	0.3570	9.69
	Total			0.5552	

**Table 2:** Removal of parabens in spiked samples by using β-CD-HMDI(a) polymer.

Sample	Methyl paraben		Ethyl paraben		Propyl paraben		Benzyl paraben	
	% removed	R.S.D. (%)	% removed	R.S.D. (%)	% removed	R.S.D. (%)	% removed	R.S.D. (%)
Deionized water (Standard)	15.38	4.54	23.21	5.41	48.44	13.46	100.00	0.00
Tap water	13.28	5.75	21.08	3.67	46.27	1.88	100.00	0.00
Drinking water	15.02	7.46	25.52	2.75	58.16	4.07	100.00	0.00
River water	14.05	7.87	24.13	3.66	56.70	1.64	100.00	0.00
Swimming pool water	13.46	6.68	22.07	8.22	49.59	8.15	100.00	0.00
Waste water effluent	12.80	6.45	20.83	4.94	46.85	3.39	100.00	0.00
Soy sauce	17.02	3.78	28.12	6.58	61.90	7.69	100.00	0.00
Mouth rinse	11.36	1.97	22.94	4.42	47.07	7.78	100.00	0.00

**Table 3:** Removal of parabens in spiked samples by using β-CD-TDI(b) polymer.

Sample	Methyl paraben		Ethyl paraben		Propyl paraben		Benzyl paraben	
	% removed	R.S.D. (%)	% removed	R.S.D. (%)	% removed	R.S.D. (%)	% removed	R.S.D. (%)
Deionized water (Standard)	33.56	1.88	51.55	2.62	95.51	1.31	100.00	0.00
Tap water	29.76	7.37	48.07	11.04	89.98	7.82	100.00	0.00
Drinking water	32.58	2.76	52.42	0.41	97.93	1.43	100.00	0.00
River water	28.56	5.92	46.28	5.30	88.29	4.31	100.00	0.00
Swimming pool water	27.09	0.27	47.35	3.59	93.34	2.89	100.00	0.00
Waste water effluent	24.24	2.41	38.77	1.61	73.63	2.21	100.00	0.00
Soy sauce	28.74	6.97	44.49	9.62	85.66	6.15	100.00	0.00
Mouth rinse	20.87	3.83	44.99	7.74	84.03	6.79	100.00	0.00
